# Pre-operative ankle-brachial index for cardiovascular risk assessment in simultaneous pancreas–kidney transplant recipients: a simple and elegant strategy!

**DOI:** 10.1186/s12893-021-01159-6

**Published:** 2021-03-22

**Authors:** Hans-Michael Hau, Nora Jahn, Max Brunotte, Tristan Wagner, Sebastian Rademacher, Daniela Branzan, Elisabeth Sucher, Daniel Seehofer, Robert Sucher

**Affiliations:** 1 Department of Visceral, Transplantation, Vascular and Thoracic Surgery, University Hospital of Leipzig, Leipzig, Germany; 2 Department of Visceral, Thoracic and Vascular Surgery, University Hospital and Faculty of Medicine Carl Gustav Carus, Technische Universität Dresden, Dresden, Germany; 3 Department of Anaesthesiology and Intensive Care Medicine, University Hospital of Leipzig, Leipzig, Germany; 4 Department of Surgery, University Hospital of Dresden, Fetscherstrasse 74, 03107 Dresden, Germany

**Keywords:** Simultaneous pancreas kidney transplantation, Graft outcome, Patient outcome, Ankle-brachial index, Diabetes mellitus, Peripheral arterial disease

## Abstract

**Background:**

Patients with insulin-dependent diabetes mellitus type 1 (IDDM1) and end-stage kidney disease (ESKD) undergoing simultaneous pancreas kidney transplantation (SPKT) are a population with diffuse atherosclerosis and elevated risk of cardio- and cerebrovascular morbidity and mortality. We aimed to investigate the feasibility of preoperative screening for peripheral arterial disease (PAD), specifically ankle-brachial index (ABI) testing, to predict peri- and postoperative outcomes in SPKT recipients.

**Methods:**

Medical data (2000–2016) from all patients with IDDM and ESKD undergoing SPKT at our transplant center were retrospectively analyzed. The correlation between PAD (defined by an abnormal ABI before SPKT and graft failure and mortality rates as primary end points, and the occurrence of acute myocardial infarction, cerebrovascular and peripheral vascular complications as secondary end points were investigated after adjustment for known cardiovascular risk factors.

**Results:**

Among 101 SPKT recipients in our transplant population who underwent structured physiological arterial studies, 17 patients (17%) were diagnosed with PAD before transplantation. PAD, as defined by a low ABI index, was an independent and significant predictor of death (HR, 2.99 (95% CI 1.00–8.87), p = 0.049) and pancreas graft failure (HR, 4.3 (95% CI 1.24–14.91), p = 0.022). No significant differences were observed for kidney graft failure (HR 1.85 (95% CI 0.76–4.50), p = 0.178). In terms of the secondary outcomes, patients with PAD were more likely to have myocardial infarction, stroke, limb ischemia, gangrene or amputation (HR, 2.90 (95% CI 1.19–7.04), p = 0.019).

**Conclusions:**

Pre-transplant screening for PAD and cardiovascular risk factors with non-invasive ABI testing may help to reduce perioperative complications in high-risk patients. Future research on long-term outcomes might provide more in depth insights in optimal treatment strategies for PAD among SPKT recipients.

## Background

Simultaneous pancreas kidney transplantation (SPKT) represents the “state of the art” treatment modality for patients with insulin-dependent diabetes mellitus type 1 (IDDM1) and end-stage kidney disease (ESKD) [1].

In recent years, increasing evidence has indicated that peripheral arterial disease (PAD) is a major healthcare burden and is highly prevalent among patients with IDDM and ESKD, as compared with the general population [2, 3]. Although several risk factors contribute to PAD, IDDM1 and ESKD represent the two major ones. The presence of PAD in patients with diabetes and ESKD-related conditions is furthermore an indicator of poor long-term outcomes, if not treated accordingly [4–6].

In fact, patients with PAD have a three- to fivefold increased risk of adverse outcomes of cardiovascular and cerebrovascular morbidity and mortality, including myocardial infarction (MI), stroke and mortality associated with coronary artery disease (CAD) [7, 8].

Additionally, patients with PAD have greater functional impairment and faster rates of functional decline than those without PAD [9, 10].

In clinical settings, the measurement of the ankle-brachial index (ABI), a non-invasive, reproducible and efficient diagnostic tool, is a widely accepted method to diagnose and predict the severity of PAD. In this context, it has been shown that ABI data are also an independent risk predictor of subsequent atheroembolic events elsewhere in the entire vascular system [11–13]. Indeed, abnormal ABI has been found to be an independent risk marker for major cardiovascular and cerebrovascular events in individuals without known pre-existing clinical cardiovascular disease (CVD) and those with established CVD [13, 14].

In this context, prospective studies have shown that ABI correlates well with overall survival [15], 16. Population-based prospective studies have furthermore shown that a low ABI was linked to an increased risk of CAD, cerebrovascular accidents (CVAs), transient ischemic attack (TIA) and peripheral vascular complications including limb ischemia/ulceration, even when age, sex and other risk factors were not taken in account [7, 13, 15–18]. However, other previous studies have found an association between high ABI and cardiovascular and cerebrovascular morbidity and mortality [8, 15, 19–21].

Coronary and cerebrovascular diseases remain the predominant causes of death in patients with diabetes before and after transplantation [22, 23].

The presence of chronic kidney disease further increases the CVD risk [24].

Assessment of perioperative cardiovascular risk and thereby the decrease in perioperative complications (e.g., cardiovascular mortality and subsequent graft failure) remains highly important in perioperative medicine; however, accurate and detailed identification of high-risk patients remains challenging. Therefore, the integration of ABI in preoperative routine testing might help improve the risk stratification and identification of patients who might benefit from special perioperative attention and, if possible, the modification of perioperative risk factors [22, 24].

Previous studies have suggested higher rates of PAD among patients awaiting kidney transplantation and have shown a reduction in risk mortality after renal transplantation [2, 25, 26]. However, little is known about the prevalence of PAD as well as the feasibility and impact of preoperative ABI testing on long-term graft and patient outcomes in SPKT transplant recipients.

This study aimed to determine whether preoperative PAD through accurate peripheral vascular evaluation by ABI testing might provide information on the risk of postoperative graft failure, death and cerebro- and cardiovascular events (independently of other CVD risk factors) and improve risk prediction for SPKT recipients.

## Methods

### *Study design and study* p*o*p*ulation*

The study protocol was approved by the local ethics committee of the University of Leipzig [AZ: Nr: 111-16-14032016]. All methods in the study were carried out in accordance with the Helsinki guidelines and declaration or any other relevant guidelines.

From a prospectively collected electronic database, we retrospectively analyzed medical data on all patients undergoing SPKT at the University Hospital of Leipzig between 2000 and 2016. We focused on the identification of patients with PAD who had undergone non-invasive vascular diagnostics, specifically ABI testing, in the pre-transplantation evaluation examination. Patients younger than 18 years, those receiving kidney transplantation alone and those receiving pancreatic re-transplantation were excluded.

### Outcome analysis

Standard demographic and clinicopathological characteristics were collected and analyzed before, at the time of and after transplantation (in the follow-up period) for each patient: the pre-transplantation data included recipient and donor characteristics such as age, sex, body mass index (BMI) and additional data including the duration of diabetes mellitus, smoking habits, time on the wait list, the duration of pre-transplantation dialysis, metabolic endocrine and lipid metabolism, information on the cardiovascular system such as the presence of coronary heart disease (coronary artery bypass grafts or stents), CVA, the type and level of PAD (ischemia, ulceration/gangrene, amputation and revascularization procedures such as bypass or angioplasty), blood pressure parameters and the number of antihypertensive agents.

### *Assessment of the cardiovascular and* p*eri*p*heral vascular system*

All patients with IDDM1 who were potential transplantation candidates underwent structured cardiovascular examinations including echocardiography and coronary angiography as a routine part of the cardiac work-up at our center before enrollment on the wait list.

For the detection of PAD, a structured vascular screening protocol including extensive physical examination (for detection of ulceration/gangrene) and symptom-oriented medical history (evaluation of claudication and chronical limb ischemia), Doppler ultrasonography such as Doppler-derived ABI testing and carotid artery duplex imaging were performed. The vessels of patients with suspected PAD were further evaluated with computed tomography/magnetic resonance angiography and/or conventional angiography, as indicated, to better define the location and extent of disease.

### PAD diagnostic tools

As a part of the pre-transplantation PAD screening, physiologic arterial vascular studies including Doppler-derived ABI measurements were performed by a well-trained vascular nurse in each patient in a quiet environment after at least 5 min of supine rest.

ABI measurement was performed with a standard hand-held continuous wave 8 MHz Doppler pencil probe and a sphygmomanometer with a 12 × 35 cm blood pressure cuff.

For ABIs, systolic blood pressures were measured and recorded in the supine position on both arms (if possible; however, not in the fistula arm), on the basis of the appearance of the pulse sound registered by the Doppler at the brachial artery as the cuff was deflated [11, 12]. Doppler measured ankle pressures were performed at the dorsalis pedis and posterior tibial artery on both legs.

The ABI was calculated by division of the systolic pressure measured in each lower limb through the systolic blood pressure of the arm with the higher pressure [12].

According to previously published consensus guidelines, participants were considered to have PAD in our analysis if they had an ABI below 0.9 and clinical symptoms (claudication/signs of limb ischemia) [27].

The ABI measurements were divided into five subcategories: normal (0.9–1.39), mild disease (0.70–0.89), moderate disease (0.40–0.69), severe disease (0–0.39) and non-compressible arteries (> 1.40) [12].

In accordance with the recent consensus guidelines, in cases of a high ABI (>1.4) or non-compressible arteries associated with medial calcification, as was evident in five of our patients with diabetes, we performed toe−brachial index (TBI) measurement and/or further noninvasive or invasive vascular diagnostic procedures for safe detection of PAD [27–29].

TBI measurement was performed as previously described in detail and was calculated by dividing the systolic pressure of the great toe by that of the brachial artery [30].

Cut-off values < 0.7 in TBI testing were defined as the diagnostic criterion for PAD [29].

### Surgical techniques/immunosuppression

The procurement and transplantation of pancreatic and kidney allografts were performed according to international standard and guidelines, as previously described [31–37]. The standard immunosuppression protocol at our center consisted of an induction therapy followed by triple maintenance medicamentous therapy, as described previously [36, 37].

### Statistical analysis

Continuous variables are reported as mean/median values with standard deviation, whereas categorical variables are presented as whole numbers and percentages (%). For analysis of baseline data, we used the appropriate statistical significance tests, including Student’s t-test, *χ*^2^, analysis of variance, Kruskal–Wallis and Wilcoxon–Mann–Whitney test.

The primary end point of this study was graft (pancreas/kidney) failure and/or death after transplantation. In this context, pancreatic graft failure was defined as insulin substitution or return to transplant, and kidney graft failure was defined as the need for dialysis or return to transplant. The secondary end points included occurrence of CVA, MI and/or peripheral vascular complications, defined as limb ischemia, gangrene, amputation or revascularization treatments (bypass surgery or angioplasty).

For the estimation of patient and graft (pancreas/kidney) survival, such as event-free survival for the secondary events after transplantation, the Kaplan–Meier method and log-rank test were used. Cox proportional hazard regression models were used to calculate hazard ratios (HR) for the primary and secondary end points in relation to PAD.

The association between PAD and the primary and secondary end points was then assessed after adjustment for known cardiovascular risk factors including recipient age, sex, recipient BMI, duration of diabetes mellitus, smoking habits, duration of dialysis, aspirin and statin use and known cardiovascular comorbidity (heart failure or CAD, CVA and MI).

If patients who had multiple ABI measurements available, we used the ABI value closest to the date of transplantation for analysis.

All data were analyzed in SPSS software (SPSS Inc., Chicago, Illinois, USA, version 21.0). A p value < 0.05 was considered statistically significant.

Note: Data from our prospectively maintained database have been previously published [36, 37]. However, these publications addressed different scientific topics and comprised different subsets of patients.

## Results

### Baseline characteristics

Between January 2000 and July 2016, a total of 101 patients undergoing SPKT at the University Hospital of Leipzig received structured evaluation for PAD, specifically ABI testing before transplantation. One patient had to be excluded from further analysis due to missing data. The mean follow-up period of the study was 101 ± 34.4 months.

Demographic and clinico-pathologic baseline characteristics between the patients with and without PAD are shown in Table 1. The mean age of the patients at the time of transplantation was 43 ± 8.9 years. Most patients were male (56%), and the mean duration of diabetes mellitus was 26.6 ± 8.6 years. The duration of pre-transplantation dialysis and the number of pre-emptive transplantations were comparable between the groups, without significant differences.

**Table 1:** Demographic and clinicopathologic characteristics of the 101 patients (with or without PAD) after SPKT

Variables	Patients without PAD (n = 84)	Patients with PAD (n = 17)	p-value
Recipient age, years	42.7 ± 9.2	44.6 ± 7.2	0.416
Recipient sex			0.308
Male	43 (51.2%)	11 (64.7%)	
Female	41 (48.8%)	6 (35.3%)	
Recipient BMI (kg/m^2^)	24.9 ± 4.3	25.8 ± 3.6	0.425
Duration of diabetes mellitus, years	26.3 ± 8.9	28.6 ± 6.9	0.330
Donor age, years	23.8 ± 11.7	27 ± 11.4	0.323
Donor BMI (kg/m^2^)	22.4 ± 3.1	25.4 ± 3.5	0.126
Donor sex			0.256
Male	52 (61.9%)	8 (47.1%)	
Female	32 (38.1%)	9 (52.9%)	
Pre-transplantation dialysis duration, months	31.0 ± 24.1	36.4 ± 24.5	0.480
Pre-emptive transplantation			0.432
Yes	17 (20%)	5 (30%)	
No	67 (80%)	12 (70%)	
Systolic blood pressure, mmHg	138 ± 17	147 ± 18	0.830
Diastolic blood pressure, mmHg	80 ± 9	85 ± 10	0.04
HbA1c pre-transplantation, %	7.8 ± 1.9	7.6 ± 1.1	0.657
Total cholesterol, μmol/l	5.3 ± 1.4	5.1 ± 1.1	0.626
Triglycerides, μmol/l	1.9 ± 1.2	1.9 ± 0.8	0.837
Limb ischemia, gangrene, amputation			0.03
Yes	3 (4%)	4 (23%)	
No	81 (96%)	13 (77%)	
CVA/TIA			0.89
Yes	4 (5%)	1 (6%)	
No	80 (95%)	16 (94%)	
Coronary heart disease			0.02
Yes	21 (25%)	9 (53%)	
No	63 (75%)	8 (47%)	
Hypertension before transplantation			0.283
Yes	74 (83%)	13 (77%)	
No	10 (17%)	4 (23%)	
Antihypertensive drugs			0.876
Assumed (n)	2.6 ± 1.3	2.4 ± 1.5	
Ever smoker			0.535
Yes	28 (33.3%)	7(41%)	
No	56 (66.7%)	10 (59%)	
Immunosuppression induction therapy			0.831
ALG/ATG	62 (73.8%)	12 (70.6%)	
IL2-RA	15 (17.9%)	4 (23.5%)	
None	7 (8.3%)	1 (5.9%)	
CNI			0.359
Tacrolimus	80 (92.5%)	17 (100%)	
Ciclosporin	4 (4.8%)	0	
AP drug			0.318
MMF	66 (78.6%)	16 (94.1%)	
SRL	14 (16.7%)	1 (5.9%)	
Multiple	3 (3.6%)	0	
None	1 (1.2%)	0	
Steroid-free after 1 year	55 (65%)	10 (59%)	0.61

In the patients in the PAD group, compared with patients without PAD, the following cardiovascular and arteriosclerotic risk factors were higher: diastolic blood pressure (p = 0.04) and the presence of known CAD (p = 0.02), such as a history of micro- and macrovascular events (limb ischemia, amputation or ulceration) (p = 0.03).

During the pre-transplantation evaluation, 17 of the 101 patients (17%) were diagnosed with PAD. Among them, 12 (12%) had a low ABI, and 5 (5%) had a high ABI. The low ABI group included mild (n = 4 patients; 4%), moderate (n = 5 patients; 5%) and severe (n = 3 patients; 3%) categories. A TBI < 0.7 and positive findings in imaging confirmed the diagnosis of PAD in the five patients with high ABI in the pre-transplantation evaluation.

Post-transplantation outcomes were measured from the time of transplantation until January 2019. Until that time point, 27 patients (27%) had secondary events including new events of cardiac disease (myocardial ischemia/ischemic heart failure) in 23 (23%) patients, events of cerebrovascular disease (TIA/stroke) in 19 (19%) patients, and further 21 patients (21%) developed peripheral vascular complications (ischemic ulceration/gangrene, amputation or revascularization). Pancreas and kidney graft failure was observed in 24 (24%) and 21 (21%) patients, respectively. Eighteen patients (18%) died after transplantation during the follow-up period.

### Outcome analysis

#### Primary endpoint

**Fig. 1:**
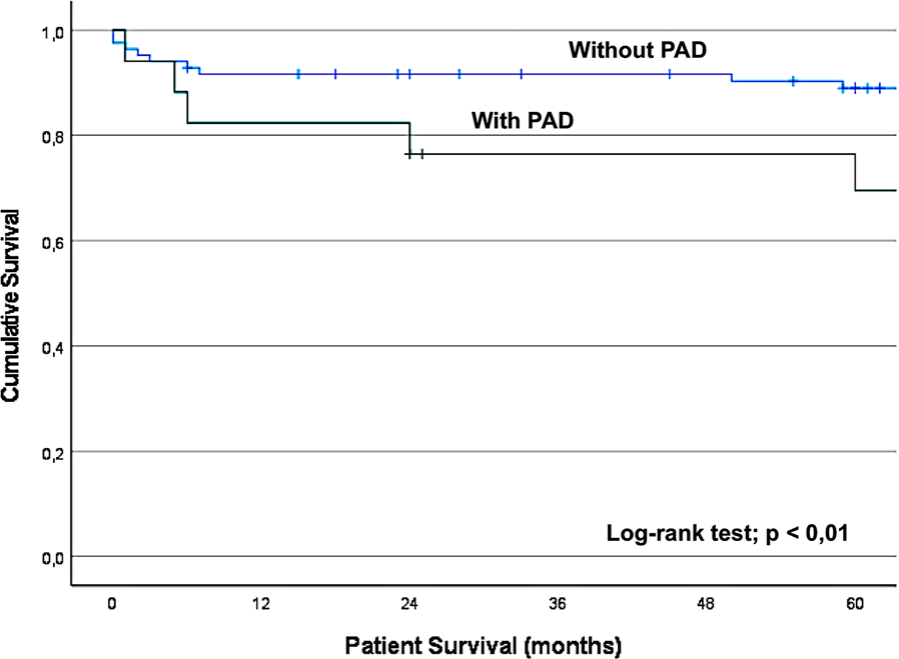
The Kaplan–Meier curve showing patient survival in the 101 patients with and without PAD, according to the ABI

**Fig. 2:**
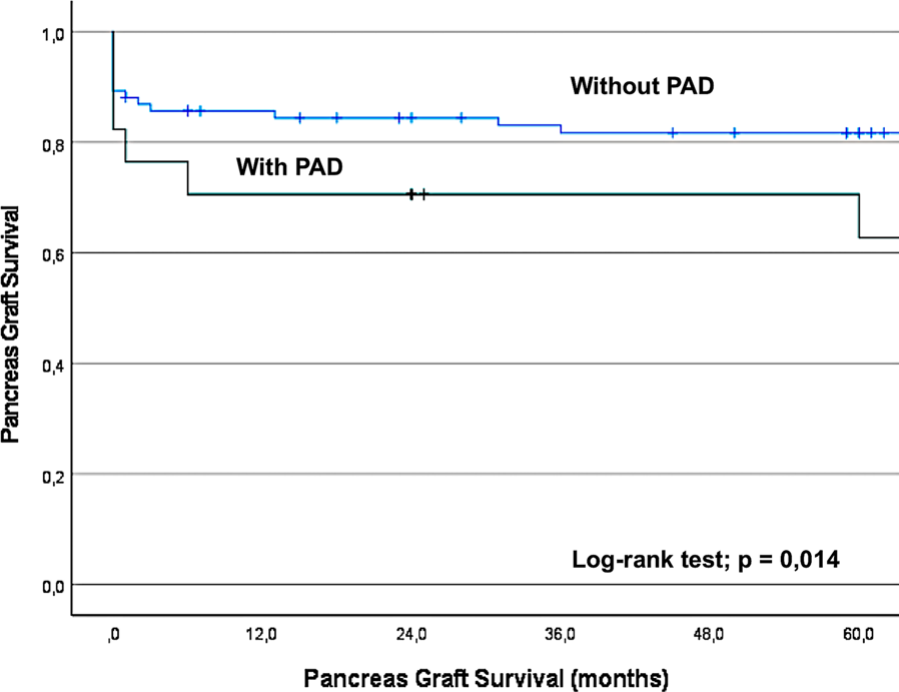
The Kaplan–Meier curve showing the pancreatic graft survival in 101 patients with and without PAD, according to the ABI

Kaplan–Meier plots for patient and pancreatic graft survival according to the presence of PAD are shown in Figs. 1 and 2.

The 5-year patient survival (89% vs. 69%, p < 0.01) and pancreatic graft survival (82% vs. 63%; p = 0.014) were significantly lower in patients with PAD.

Tables 2, 3 and 4 show the results of Cox regression analysis for the primary and secondary endpoints, with adjustment for the recipients’ confounding variables, including age, sex, BMI, duration of diabetes mellitus, smoking habits, duration of dialysis, aspirin and statin use, and known cardiovascular comorbidity (heart failure or CAD, CVA, MI, peripheral vascular complications).

**Table 2:** Cox regression analysis for death in SPKT recipients

Variable	HR	95% CI	p-value
PAD	2.99	1.00–8.87	0.048
Recipient age (years)	0.98	0.89–1.07	0.608
Gender	1.04	0.30–3.53	0.947
Diabetes duration (years)	1.10	1.02–1.19	0.012
Number of antihypertensive drugs	1.17	0.79–1.72	0.438
Aspirin	0.537	0.14–2.06	0.365
Statin	0.84	0.23–3.13	0.800
Cardiovascular comorbidity	4.29	1.33–13.81	0.015
Smoking history	1.84	0.58–5.82	0.300
Dialysis duration (months)	1.01	0.99–1.03	0.212

**Table 3:** Cox regression analysis for pancreatic graft failure in SPKT recipients

Variable	HR	95% CI	p-value
PAD	3.55	1.16–10.87	0.026
Recipient age (years)	1.01	0.95–1.08	0.734
Gender	2.71	0.94–7.82	0.065
Diabetes duration (years)	1.00	0.93–1.08	0.996
Number of antihypertensive drugs	1.61	1.08–2.40	0.019
Aspirin	0.80	0.27–2.44	0.701
Statin	1.90	0.61–5.92	0.265
Cardiovascular comorbidity	3.70	1.28–10.77	0.016
Smoking history	1.17	0.32–4.29	0.817
Dialysis duration (months)	0.99	0.96–1.01	0.303

**Table 4:** Cox regression analysis for Secondary events in SPKT recipients

Variable	HR	95% CI	p-value
PAD	2.90	1.19–7.04	0.019
Recipient age (years)	1.02	0.96–1.08	0.483
Gender	1.44	0.58–3.57	0.433
Diabetes duration (years)	1.0	0.94–1.06	0.908
Number of antihypertensive drugs	1.15	0.86–1.53	0.348
Aspirin	1.13	0.44–2.95	0.797
Statin	0.56	0.23–1.35	0.195
Cardiovascular comorbidity	2.69	1.11–6.51	0.028
Smoking history	1.10	0.39–3.07	0.864
Dialysis duration (months)	1.01	0.99–1.03	0.510

We found that PAD, defined by a low ABI, was an independent and significant predictor and risk factor of death (HR, 2.99 (95% CI 1.00–8.87), p = 0.049) and pancreatic allograft failure (HR, 4.3 (95% CI 1.24–14.91), p = 0.022). However, no significant differences were observed for kidney allograft failure (HR 1.85 (95% CI 0.76–4.50), p = 0.178). A normal ABI was associated with better patient survival (HR, 0.34 (95% CI 0.11–0.99, p = 0.049) in our patient cohort.

**Fig. 3:**
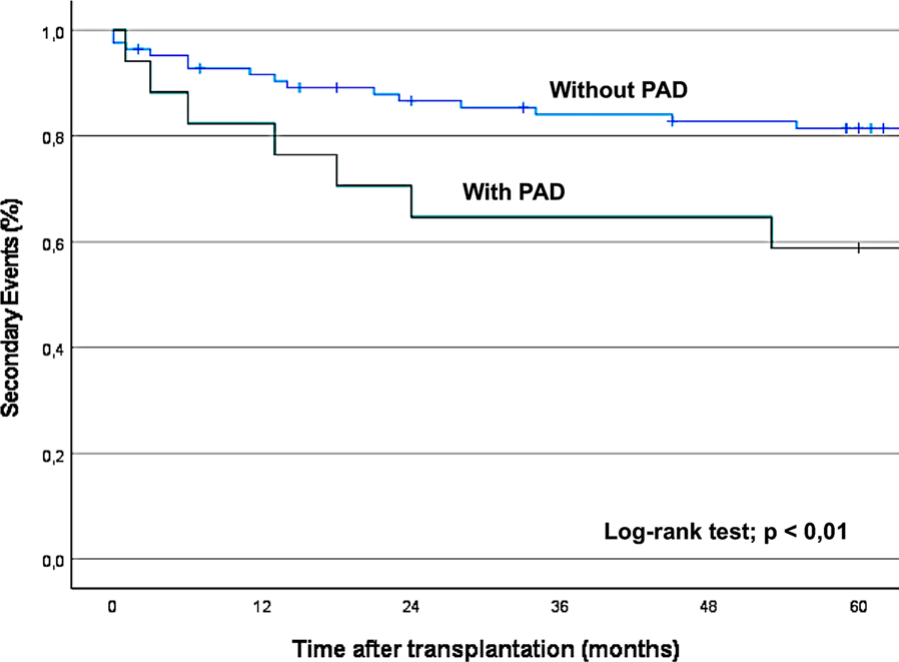
The Kaplan–Meier curve showing the incidence of secondary outcomes (MI, CVA, limb ischemia, amputation and gangrene) in patients with and without PAD, according to the ABI

Patients with PAD were at greater risk of pancreatic graft failure, secondary outcomes and death (Figs. 1, 2 and 3) than those without PAD, as defined by a normal ABI.

#### Secondary endpoint

With regard to secondary outcomes, a low ABI was an independent and significant predictor of early MI, stroke, limb ischemia, gangrene and amputation (HR, 2.90 (95% CI 1.19–7.04), p = 0.019) (Table 4, Fig. 3). In addition, cardiovascular morbidity (HR 2.69; p = 0.028) was an independent significant predictor of secondary events (Table 4).

## Discussion

It’s a scientific fact that the presence of IDDM1 and ESKD are strongly related to accelerated development of atherosclerosis, and further development of PAD [2, 8, 17]. Patients with PAD have a significantly higher risk of cardiovascular and cerebrovascular events, resulting in significant decrease of quality of life and functional worsening [15, 38, 39]. However, data are limited regarding the prevalence and clinical effects of PAD, specifically on the basis of pre-transplantation ABI testing, on the long-term outcomes in SPKT recipients.

The detection of arteriosclerosis at early stages through adequate and non-invasive preoperative screening and the consequent initiation of optimal preventive medical treatment may help decrease perioperative cardiovascular mortality before transplantation [40]. In this context, the clinical assessment of resting ABI is an accurate, simple and non-invasive diagnostic test to assess the arterial vessel system of the lower extremities, and is additionally a reliable predictor of the presence of lower extremity PAD [12, 19].

The ABI is also an indicator of atherosclerosis at other vascular sites, and it can serve as a prognostic marker of cardiovascular events and functional impairment even in the absence of PAD symptoms [7, 9, 13].

However, the conditions and comorbidities associated with media calcification and vessel stiffness, such as IDDM, ESKD and advanced age, can lead to falsely elevated or normal pressures [2, 28, 40]. Under these circumstances, the measurement of TBI is useful, because the toe vessels are relatively less susceptible to vessel stiffness, and TBI can help provide a more accurate determination of vascular disease in this setting than ABI alone [29].

In our pre-transplantation screening algorithms, the vascular PAD diagnostic consisted of structured arterial physiologic testing, including Doppler-derived ABI or alternatively TBI testing, ultrasonography, patient history and clinical examination.

TBI testing is performed for diagnostic assurance in patients with elevated ABIs and expected vessel stiffness due to relevant comorbidities [41].

In cases of functional symptoms, further noninvasive and invasive physiologic examinations were performed, including pulse wave, Doppler wave and computed/magnetic resonance angiography and walking tests on a treadmill. In this current study, we sought to address the value of ABI testing in a large population of SPKT recipients.

In the pre-transplantation evaluation examinations of SPKT recipients, we found that PAD was identified by a structured lower arterial extremity physiologic evaluation in almost one-fifth of our patients over a study period of approximately 20 years. This finding is consistent with the few previous studies that have found an increased prevalence of PAD in patients with diabetes with ERSD waiting for kidney and/or pancreas transplantation, as compared with patients without these risk factors [25, 42, 43].

Furthermore, we observed that PAD defined by a low ABI was an independent and significant predictor of postoperative patient death, pancreatic graft failure and postoperative cardio- and cerebrovascular events (MI, stroke or peripheral vascular complications) in SPKT recipients. This result was independent of other cardiovascular comorbidities and the number of years of dialysis. Our findings support the use of ABI or accurate preoperative PAD testing for predicting mortality and graft failure in individuals with PAD, independently of other known cardiovascular risk factors.

Recent reports have demonstrated that a successful SPKT that leads to euglycemia can slow the progress of macrovesicular disease, as described in PAD [42–44].

In contrast, according to previous studies from renal transplant recipients, the presence of PAD is associated with an increased risk of allograft failure, as was also seen in our study [6, 26]. However, the pathophysiology underlying the increased risk of graft failure in patients with PAD is not well understood, although factors such as the presence of toxins, arteriosclerotic disease and the inflammatory state of PAD may also play roles [2]. Nevertheless, one explanation may be that the transplantation itself and the use of immunosuppressive medicaments exacerbates the pre-existing risk factors that lead to atherosclerosis or the development of new cardiovascular risk factors [2].

However, despite recent excellent advances in treatment, patients with SPKT, IDDM and ESKD-related conditions tend to have high rates of cardio- and cerebrovascular complications, and CVD remains the leading cause of mortality in SPKT recipients with functioning grafts, as also seen in the current study [23, 24, 45].

Despite of the immanent perioperative cardiovascular risk, successful SPKT offers the best known protection against the progression of CVD and future cardiovascular events [23, 46–48]. Previous studies on kidney–pancreas transplantation have demonstrated the importance of low-risk but highly sensitive screening strategies for major adverse cardiovascular events [22, 24, 49]. Despite these findings, an optimal strategy for cardiovascular risk and postoperative graft outcome assessment in these patients remains lacking. To date, published approaches vary from evaluating patients with different risk scores to screening all patients being considered for SPKT [49–51].

Nevertheless, coronary angiography, an invasive and cost-intensive technique, should be applied only in high-risk patients with a long history of diabetes, severe peripheral or coronary vascular disease or a history of acute myocardial infarction [52, 53].

Therefore, the preoperative assessment of all patients undergoing SPKT by coronary angiography does not appear to be feasible; however, because transplantation in cardiovascular high-risk patients is increasing, a non-invasive but sufficiently sensitive stratification strategy is needed to assess perioperative cardiovascular risk as well as transplant outcomes.

However, a consensus is lacking regarding the best assessment and optimization strategy for cardiovascular risk and transplant outcomes.

In the current study, we therefore aimed to verify the value of screening for PAD through pre-transplantation ABI testing to identify cardiovascular high-risk patients who were eligible for further invasive assessment, as well as specifically modified preoperative risk factors and perioperative protective strategies.

We found that ABI testing is an inexpensive and easily applicable assessment tool during preoperative screening of patients eligible for SPKT.

### Limitations

First, this study was limited by its retrospective nature and small sample size, particularly in the ABI subcategories (‘low-ABI’ and ‘high-ABI’ patients) in both the pre-transplantation and post-transplantation ABI results; the sample size was too small for further analysis or drawing conclusions.

Second, in few SPKT recipients (n = 6; 5.9%), vascular exams were performed quite some time before the transplantation (specifically during pre-transplantation screening examinations for placement on the wait list), and these patients were subsequently categorized as normal controls. However, if peripheral vascular disease and arteriosclerosis might have progressed while the patient was on the waiting list without additional documentation this might have created a small bias. In other words, this bias may have led to an underestimation of PAD at the time of transplant and consecutive outcome analyses. Ideally, all patients should have their vascular diagnostics performed within a year after transplantation.

Third, the results of ABI testing in our patient cohort with IDDM and ESKD must be interpreted carefully, because both comorbidities could lead to increased vascular calcification, thus resulting in vessel stiffness and/or non-compressible vessels [2, 28]. The incompressibility of the vessels could lead to elevated ABIs > 1.4 or falsely normal ABIs. In this setting, the use of an additional TBI testing, toe pressure measurement or Doppler waveform data, which were conducted in our analysis in these patients, would have helped further characterize the patients with an ABI > 1.4, because toe vessels are relatively less affected by calcification [11, 29].

Fourth, the natural history of PAD involves a decrease in ABI over time. However, why the ABI would increase in some patients and decrease in others after transplantation is not well understood. The reason for these findings remains unclear, and the principal patterns of causation should be further evaluated in future prospective studies.

### Conclusions

In conclusion, we demonstrated that ABI evaluation combined with TBI testing in unclear cases is a valuable, inexpensive and feasible assessment tool for accurate examination of perioperative cardiovascular risk in patients undergoing SPKT. We showed that PAD associated with low and high ABIs predicted higher mortality, pancreatic graft failure and poorer cardiovascular outcomes. With the information gained from preoperative ABI testing, patients at high-risk for perioperative cardiovascular complications and simultaneous graft failure can be identified so that further invasive examination and, if possible, reduction of preexisting risk factors can be initiated.

Further research, ideally in large randomized and controlled multicenter trials, are needed to evaluate the use of ABI testing in pre-operative PAD screening in high-risk patient populations. Moreover, future research should focus on the evaluation of functional capacity or walking distance as a comparative tool for ABI testing, to establish the role of ABI in further perioperative risk assessment in high-risk cardiovascular patients and to identify different ABI subcategories for optimized risk stratification.

## Data Availability

Our database contains highly sensitive data that may reveal clinical and personnel information about our patients and lead to their identification. Therefore, according to organizational restrictions and regulations, these data cannot be made publicly available. Nevertheless, the datasets used and/or analyzed in the current study are available from the corresponding author upon reasonable request.

## Abbreviations

SPKT: Simultaneous pancreas–kidney transplantation
IDDM1: Insulin-dependent diabetes mellitus type 1
ESKD: End-stage kidney disease
PAD: Peripheral arterial disease
ABI: Ankle-brachial index
CVD: Cardiovascular disease
CAD: Coronary artery disease
CVA: Cerebrovascular accident
TIA: Transient ischemic attack
MI: Myocardial infarction
BMI: Body mass index
CABG: Coronary artery bypass graft
CTA: Computed tomography angiography
MRA: Magnetic resonance angiography
TBI: Toe-brachial index
DSO: Deutsche Stiftung Organtransplantation
CNI: Calcineurin inhibitor
MMF: Mycophenolate mofetil
SRL: Sirolimus
ATG: Antithymoctye globulin
IL2-RA: Interleukin-receptor-antagonist
